# Pivot Teeth, Crowns, Etc.

**Published:** 1888-12

**Authors:** W. St. George Elliott


ARTICLE V.

### PIVOT TEETH, CROWNS, &C.

BY W. ST. GEORGE ELLIOTT, M. D., D. D. S.

[Extracts from Lectures at National Dental College on Operative Dental Surgery.]

Pivot teeth have probably been used to a limited extent for several hundred years. We are certain that they were known over a century ago, as Woofendale has left us the record as far back as 1783. At the same time, they must have been seldom met with up to a comparatively recent date. Attention has only been called prominently to the matter within the last twenty-five years.

Human teeth were solely relied upon in the days anterior to the introduction of porcelain teeth, and are occasionally to be met with in our own time. There is some temptation to use them, on account of their natural appearance, but the decay which so generally sets in, added to the disagreeable odor that they produce, has properly banished them for ever.

The earliest form of pivot, or pin, was that of wood. This material, while for many years extensively used, is now only occasionally met with, it having been replaced by the more desirable metal substitutes. When wood is preferred, compressed second-growth hickory is found to be the best, and the pin used should be one-eighth of an inch in diameter and half-an-inch long. The old-fashioned pivot crowns in America were shaped like the natural tooth above the gum, and had a hole in the axis which, however, did not pass through, as in tube teeth. The wooden pin was forced into the crown, and fitted while dry into the hole prepared in the root; reliance was placed upon the moisture to swell the wood, and thus make the tooth firm.

In some mouths wooden pivots have proved fairly durable, but in the majority they are of little value. I remember seeing a case while practicing in Callao (Peru) in which the wooden pin had been in place for fifteen years and was still perfectly serviceable.

Soon after wooden pivots came into general use attention was given to metal substitutes, as well as to a combination of the two—a wire centre in a wooden pivot (White, 1861)—and from that time down to the present the different kinds of pins, screws, tubes, caps, &c., that have come before us is something remarkable; probably not less than one hundred and fifty have been published. I cannot, however, go into all the varied forms, even if I had accessible data to draw from, but will merely outline some of the more prominent, mainly for the purpose of preventing their being re-invented and again offered to the profession as something new and valuable.

I shall try to classify and arrange some of the many forms; but many absolutely refuse to fall into line, and must be described separately:

Wood pin or pivot in nerve canal, with and without cement packing. (Woofendale, 1783; Willard, 1852; Muse, 1853; McQuillen, 1863.)

Wood pin in tube forced into canal, with and without cement. (Old Practice, 1876.)

Wood pin in tube, screwed into root.

Vulcanite pin, with and without tube and packing.

Vulcanite pin with wire centre. (Richardson, 1862.)

Metallic pin soldered to plate tooth and forced into root, with or without cement. (Van Marten, 1874; Flagg, 1865.)

Metallic pin vulcanized to vulcanite tooth forced into root, with or without cement. (Richardson, 1862.)

Metallic pin cemented in root, and projecting into and cemented to crown. (Bonwill, 1880.)

Metallic pin, split or otherwise, fitted to tube in root, pin fastened to crown. (Balkill, 1875.)

Metallic pin soldered to cyl. cap, cemented into root. (Buttner, 1883.)

Metallic screw in nerve canal, with and without cement, with nut on projecting portion. (Bonwill, 1875.)

Metallic screw of silver, screwed into gold-filled root, crown attached to same by Wood's metal. (Latimer, 1865.)

Metallic screw in tube, tube tapped inside or out, or both. (Richmond, 1879.)

Metallic screw in canal, with or without cement.

Metallic screw through crown into wood in root. (Foster, 1879.)

Metallic screw through vulcanite, backing in root. (Register, 1875.)

Metallic screw in wood in root, split and projecting; wood wedge and ferrule and pivot crown. (Wetherby, 1866.)

CROWNS:

All gold, swedged, with cross bar, cement packing. (Morrison, 1869; Beers, 1873.)

All gold, low cap to stump, screws through into root, gold crown fitted over. (Talbot, 1880.)

All gold, low cap to stump, swedged cusps, space between filled with melted gold. (Rollo Knapp, 1887.)

All gold, struck up from sheet metal, without joint. (Reiner, 1885.)

PORCELAIN-FACED CROWNS:

Cap to stump, plate tooth contoured with gold, cemented pin in root. (Richmond, 1879.)

Cap to root, sheet metal sides, centre filled in with melted gold. (Rollo Knapp, 1886.)

Hollow crown, with porcelain front let into same, and held by cement, &c.

All-gold crowns were introduced to the profession by Dr. Morrison, of St. Louis, about 1869, but they were not generally known, or, where known, not fully appreciated, until within the last ten years. They are, in my opinion, one of the most valuable means at our disposal for rendering broken-down and otherwise unserviceable molars useful.

Briefly, they are hollow crowns made of No. 20-carat gold, and shaped like the original tooth. They may be made in a number of ways, but I prefer the following: The root is prepared by cleaning and stopping the canals in a thorough manner, for which purpose I use liquid and solid gutta perchas. If the walls are sufficiently thick, undercuts are made in them to anchor the cement filling; if they are not strong, pins are fitted into the canals, either at a sitting in advance of the final completion, or at the same time. The standing walls, if any, are cut away sufficiently, so that they do not interfere with the fitting of the crown or with its articulation. An impression is now taken of the tooth in gutta percha, and the bite in Stent, and a model cast in Spence metal. Upon this model the band is fitted and soldered. When this is done, a suitable cap is selected, fitted into the band with wax, and the whole tried in the mouth, and both band and cap fitted, the one to the gum, the other to the antagonizing molar. The whole is then removed, invested in plaster and sand, and soldered with 18 carat solder.

A staple of gold wire, or other suitable mechanical means of anchorage, is now soldered in the inside of the cap, which, after polishing, is ready to be placed in the mouth.

The cavity and tooth having been thoroughly dried by means of Japanese paper and the hot-air syringe, and the pins placed in the canals, the cavity and the crown are filled with oxyphosphate cement, the crown placed in position and bitten upon to see that the articulation is correct. The cement in excess is found squeezed out beside the gum; this is removed, the parts kept dry for a few minutes, and the operation is complete.

There are several advantages in this mode of procedure. It takes less time and is much easier to the operator and the patient than if the band were fitted entirely in the mouth. It is vastly simpler and much less expensive than the Rollo Knapp process, which is as follows—

The tooth is cut down almost to the gum, on this a cap is fitted not unlike the lid to a bandbox: this is a difficult operation and takes much time. Upon this cap is built a wax tooth; this is artistically carved and properly articulated; it is then removed from the mouth. An impression of the top of this wax tooth is taken in pumice stone and glycerine, and into this is poured zinc. With a die thus made and a block of lead the cap or cusps are struck up from thin sheet gold. The cusps, after trimming, are placed upon the wax model and the sides are covered in by sheet gold, leaving an opening. The sheet metal connects the gold cap or cusps with the shallow cap intended to cover the root.

The tooth is now invested in plaster and marble dust, except the side uncovered, and when dry it is heated up by the Knapp blow-pipe, which, having a small and pointed flame, melts the bits of gold and solder that are put in the hole, the melted metal taking the place of the wax.

The blow-pipe referred to uses nitrous oxide under pressure, as in the ordinary fluid form, together with common illuminating gas. The resulting flame is small and pointed, and is quite efficient for the purpose specified. The consumption of nitrous oxide is about a quart in six minutes, a gallon thus lasting twenty-four minutes, depending, of course, on the size of flame; this cannot be considered expensive; and yet those who can obtain Bine's oxygen, as we have it in London, are much better off, as the cost is 2½d. a foot as against 8d., or less than one-third. The advantage in the nitrous oxide is that it is always to be found in every village. But it is quite unnecessary to employ any special apparatus as a blow-pipe; merely attach a tube to the illuminating gas, and another to your nitrous oxide bottle, and connect the two at a common mouth blow-pipe. I have thus used them for a year without trouble.

In the latter process about double the gold is required and more than double the time, as five hours are considered by Dr. Knapp as necessary, while the former method—which is not the invention of any one person—takes but two hours. We are indebted to Dr. Starr for the means referred to of rapidly swedging the caps, in excellent imitation of the natural cusps, &c.

It is desirable, although not a necessity, that the sides of the hollow crown just described should be rounded or contoured. This is done after the band has been soldered together. The central portion of the band is, by the aid of the contouring pliers, bulged, or rounded outwards, so that a horizontal line drawn through the centre of the tooth is longer in the centre than at the cervicle edge.

For many reasons it is considered an advantage to have the edges of the crown fit snugly around the neck of the tooth, but I have never found any gum irritation resulting from even a poorly adapted crown. The edge should press lightly against the gum all round, but not sufficiently to cause discomfort to the patient.

### PIVOT TEETH.

Pivot teeth, as ordinarily understood, mean all porcelain crowns as applied to the front teeth; the general word crown is, however, displacing the older pivot. While lecturing on the subject last year, you may remember, I described some seventy modes of attachment. In the earlier part of this lecture I attempted to classify them, mainly by the character of the attachment. As they are only of interest from a historical point of view, we will not again refer to them, except to the few whose claim upon us cannot be summarily dismissed. The first special crown largely introduced since the days of the wood pin is the Bonwill, 1878; this is not the screw and nut crown of the same prolific inventor, but a materially different one. It consists of a porcelain crown with conical hollow interior, connecting with a small hole in lingual aspect. The root is filled with amalgam, a stout pin forced into it, the crown filled in the same way and forced over the projecting pin in root. When hard this tooth is generally very suitable, but it is often detached before the amalgam has sufficient time to harden, much to the discomfort of all concerned.

Personally I prefer the earlier screw and nut. With this I have met with much success. My plan of procedure is as follows. The root is disinfected and coned out in excess of the requirements of the pin; it is then undercut with a wheel bur and thoroughly dried with hot air; the cavity is then filled with oxyphosphate, and while still quite soft, the crown with its screw and nut in position, is introduced and moved into proper articulation. At a subsequent sitting, if there is no hurry, I take off the crown by releasing the nut, remove the oxyphosphates from the crevice, undercut and fill with amalgam, refit the crown and put some amalgam over the nut, or what is better, put on a second nut over the first—this generally prevents the loosening and falling off of the nut, which is the weak point in this mode of attachment.

Perhaps the easiest and the most satisfactory form of pivot is that where the pin has been baked into the body of the tooth, as in the one form of Richmond, the Logan, Brown, &c. The cavity is fully coned out and undercut and filled with oxyphosphate, and the crown introduced. As the crown covers the cement, and largely protects it from the action of the saliva, fair durability may be expected of it.

The manifest objection to this and all other special forms is the necessity of an enormous number to match from. Depots do not care to carry a large stock, and if the chances of matching are poor, one must have recourse to some other plan. It is for this reason I generally make my own pivots, using a plate tooth backed and contoured with gold and pin attached, or plate tooth backed and contoured with gold, with a hole in centre for the screw and nut.—*Dental Record.*

